# The transcriptomic basis of tissue‐ and nutrition‐dependent sexual dimorphism in the beetle *Onthophagus taurus*

**DOI:** 10.1002/ece3.1933

**Published:** 2016-02-12

**Authors:** Cristina C. Ledón‐Rettig, Armin P. Moczek

**Affiliations:** ^1^ Department of BiologyIndiana University915 E. 3rd StreetBloomingtonIN47405USA

**Keywords:** Condition dependence, developmental plasticity, genic capture, polyphenism, sex‐bias, sexual selection

## Abstract

Sexual dimorphism accounts for a large fraction of intraspecific diversity. However, not all traits are equally sexually dimorphic; instead, individuals are mosaics of tissues that vary in their ability to exhibit dimorphism. Furthermore, the degree of a trait's sexual dimorphism is frequently environment‐dependent, with elaborate sexual dimorphism commonly being restricted to high nutritional conditions. Understanding the developmental basis and evolution of condition‐dependent sexual dimorphism can be critically informed by determining – across tissues and nutritional conditions – what sex‐biased genes are deployed and how they interact and translate into functional processes. Indeed, key theories concerning the evolution of condition‐dependent sexually dimorphic traits rest on assumptions regarding their developmental genetic underpinnings, yet, have largely gone unexamined by empirical studies. Here, we provide such evidence by investigating the transcriptomic basis of tissue‐ and nutrition‐dependent sexual dimorphism in the bull‐headed dung beetle *Onthophagus taurus*. Our findings suggest (1) that generating morphological sexual dimorphism requires sex‐biased gene expression in and developmental remodeling of both sexes, regardless of which sex exhibits externally visible trait exaggeration, (2) that although sexually dimorphic phenotypes are comprised of traits underlain by independent repertoires of sex‐biased gene expression, they act similarly at a functional level, and (3) that sexual dimorphism and condition‐dependence share common genetic underpinnings specifically in sexually‐selected traits.

## Introduction

**Figure 1:**
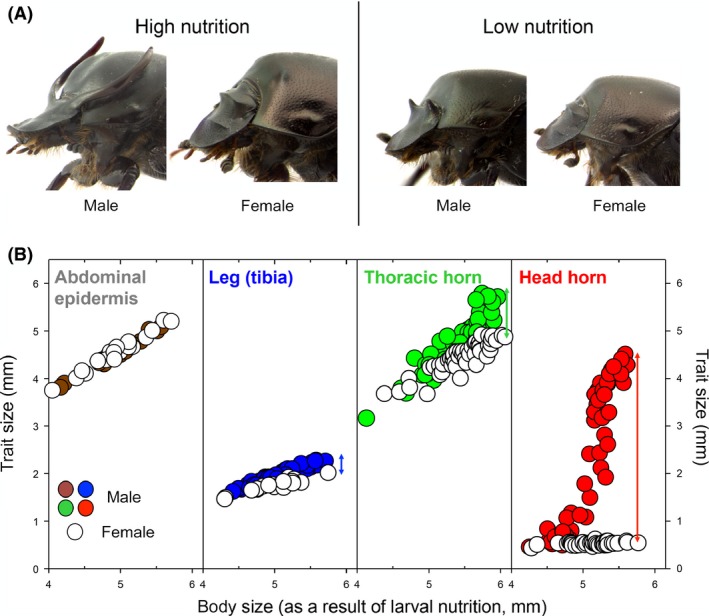
Sexual dimorphism is a function of nutrition in *Onthophagus taurus*. (A) Closeups of male and female adult *O.taurus* that experienced high (left) or low (right) larval nutrition. Note that sexual dimorphism is by far most pronounced under high nutritional conditions. (B) Male and female scaling relationships between body size, as determined by larval nutrition, and the four focal traits examined in this study. Traits differ in the degree to which sexual dimorphism is influenced by nutrition. Male and female traits do not diverge with increasing nutrition (abdominal epidermis), or alternatively diverge mildly (legs), moderately (thoracic horns), or dramatically (head horns) respectively. Images and data modified after Kijimoto et al. (2014).

Sexual dimorphism – the divergence of phenotypes between males and females of the same species – often accounts for the greatest breadth of intraspecific variation in the natural world, despite the fact that sexes share, to a large degree, the same genetic information (Ellegren and Parsch 2007). At the same time, sexually dimorphic traits are often those most greatly modified in their expression by external conditions, in particular nutrition, with sexual dimorphism often being most pronounced under high nutritional conditions (reviewed in Price et al. 1993; Fig. 1A). Lastly, not all traits within an individual are equally sexually dimorphic; instead, sexual dimorphism is highly variable among traits, such that individuals can be viewed as mosaics of parts that vary in their ability to exhibit dimorphism.

Because the two sexes of a species share virtually the same genome, it is thought that the majority of sexually dimorphic phenotypic variation must arise through differential gene expression (Ellegren and Parsch 2007). Although the genetic mechanisms that *initiate* sexual dimorphism at a morphological level are well‐studied in model organisms (Williams and Carroll 2009), much less is known about the transcriptional dynamics that underlie condition‐dependent development of morphological sexual dimorphism – or the tissue‐specificity of such dimorphism – beyond the examination of select candidate pathways. For instance, Emlen et al. (2012) found that the insulin signaling pathway – widely implicated in coordinating growth with nutritional conditions – plays a key role in the sexually dimorphic, condition‐dependent and tissue‐specific development of head horns in rhinoceros beetles. Similarly, a study utilizing a temperature‐sensitive insulin receptor mutation in *Drosophila* demonstrated that variation in this pathway governs allometric relationships between organs; whereas a reduction in insulin‐signaling (a proxy for nutritional conditions) caused a reduction in wing, maxillary palp and overall body size, it left genitalia unaffected (Shingleton et al. 2005). Furthermore, the *doublesex* pathway, typically implicated in sexual differentiation, has been found to mediate nutrition‐dependent trait exaggeration in mandibles of male stag beetles (Gotoh et al. 2014) and the head horns of male dung beetles (Kijimoto et al. 2012). Together, these studies highlight the modularity of certain pathways and their potential for generating novel and diverse condition‐dependent sexually dimorphic traits.

However, relying solely on candidate pathways potentially biases our perspective on how most genes behave in response to genetic sex and nutritional context, a view better approached through unbiased transcriptome‐wide approaches. Yet, thus far only a modest number of transcriptomic studies have simultaneously addressed genome‐wide responses to sex and nutrition in multiple tissues. Existing work includes investigations of condition‐dependent sex‐biased gene expression in fly populations (Wyman et al. 2010) that, however, was unable to resolve the potential significance of tissue‐specificity on condition‐dependent sexual dimorphisms, as well as several studies investigating tissue‐specific sex‐biased gene expression in diverse systems including chicken (Ellegren et al. 2007), mosquitoes (Baker et al. 2011), mice (Yang et al. 2006), and turkeys (Pointer et al. 2013) that, however, all focused on tissues that were either not condition‐dependent in their development and/or not obviously sexually dimorphic at a morphological level. Thus, our understanding of developmental genetic mechanisms that underlie the dramatic morphologies emerging from the interactions between genetic sex, tissue identity and condition, remains limited.

Here, we address the transcriptomic basis of tissue‐specific and condition‐dependent sexual dimorphism in the bull‐headed dung beetle *Onthophagus taurus,* named so for a pair of enormous, sexually selected head horns used as weapons in male combat and expressed only in large, high nutrition males. In this species, as is typical for a wide range of organisms, males and females differ substantially in tissue‐specific growth responses to nutritional variation. We focused our analysis on four tissues ‐ abdominal epidermis, legs, thoracic horns, and head horns ‐ that under low larval nutrient availability exhibit similar growth dynamics in males and females, such that the resulting adults appear largely sexually monomorphic. Under high nutrition, all female tissues exhibit growth increases roughly proportional to each other, whereas the corresponding male tissues exhibit a wide range of nutritional responsiveness, from negligible (abdominal epidermis) to modest (legs), to substantial (thoracic horns) and finally explosive and nonlinear (head horns). Combined, the striking differences in growth responses among male tissues generate both a remarkable sexual dimorphism as well within‐male polyphenism at high nutrition (Fig. 1).

We utilize the diversity of sex‐specific growth responses to nutritional variation across these four tissues to address basic questions regarding the genetic and developmental mechanisms underlying condition‐dependent, sexually dimorphic phenotypes. Specifically, we sought to address the following fundamental questions: (1) Does sex‐biased gene expression co‐vary with the magnitude of sexually dimorphic trait exaggeration observable on a morphological level for a given trait, sex and nutritional context? (2) Is sex‐biased gene expression trait‐specific or shared across multiple tissues, and does this depend on nutritional context? And lastly, (3) How is condition‐dependent sex‐biased gene expression translated into sex‐biased functional processes within single and across multiple tissues?

At the same time we use our approach to address specific predictions derived from current evolutionary models aimed at understanding the origin and diversification of condition‐dependent secondary sexual traits. First, we test a key prediction emerging from sexual selection theory (Levine and Tjian 2003; West‐Eberhard 2003; Carroll 2008; Wyman et al. 2012) that the sex with the most dramatic trait elaboration should also express the most numerous sex‐biased genes; that is, morphological complexity should scale with transcriptional complexity. Furthermore, we determine whether sex‐biased gene expression is generally tissue‐specific in order to alleviate pleiotropic effects in other tissues (Hodgkin 1998; Meisel 2011), or broadly shared across traits to enable integrated and coordinated transcriptomic responses to sex and nutrition (Cheverud 1996; Wagner 1996), two competing predictions derived from alternative models of the evolution of conditional development. Finally, we test a key prediction of the genic capture hypothesis – an explanation for the presence of genetic variation in sexually selected traits – that traits under sexual selection, such as male head horns, should depend on the expression of genes that contribute to both sexual dimorphism *and* nutrition‐responsiveness (Rowe and Houle 1996).

## Methods

### Experimental design ‐ overview

This study sought to characterize the transcriptomic differences among four different body regions in male and female *O. taurus* at both high and low nutritional conditions. To do so, we executed a multifactorial microarray experiment using a custom Nimblegen gene expression array (Roche Nimblegen, Inc., Madison, WI) with probes to 42,010 contigs representing at least 10,000 *Onthophagus* genes (Choi et al. 2010). Most contigs were represented by three probes, whose signals were averaged in the subsequent analysis (see below).

The data used in this study were generated at the same time as those utilized by Kijimoto et al. (2014; details on the experimental design, microarray platform, and statistical analyses can be found therein), but were derived from completely different microarray contrasts. Whereas the previous study analyzed eight microarray hybridizations between two nutritional conditions for homologous tissues within sexes (e.g., large male head horns vs. small male head horns), the current study analyzed eight separate microarray hybridizations between two sexes for homologous tissues within nutritional conditions (e.g., large male head horns vs. large female head horns). Because microarrays compare *relative* levels of gene expression, a microarray comparing nutritional morphs (such as those used in Kijimoto et al. 2014) can only reveal gene expression changes *within the same sex across nutritional conditions*, and as such is inappropriate for addressing how the same genes may behave across sexes, and *visa versa* for sex‐biased arrays. How sex‐biased genes behave across nutritional conditions can be, however, ascertained by finding the intersection of these two, independent datasets (see section E, “Comparison of Nutrition‐Dependent and Sex‐Biased contigs”).

For each of our eight contrasts (four tissues × two nutritional conditions), pools of contigs whose variation in expression levels were significantly explained by sex were determined using Ordinary Least Squares. We use the term “sex‐biased” to reflect significant differential expression between homologous male and female tissues under a particular nutritional condition, and the term “degree” to indicate the number of contigs in an experimental group that exhibit significant sex‐biased expression. Lastly, we refer to male‐biased genes as those more greatly expressed in males relative to females, and female‐biased genes as those more greatly expressed in females relative to males, irrespective of absolute expression levels. Below, we briefly summarize the most important methodological steps.

### Animal husbandry

Beetles were collected and reared as described in previous studies (Shafiei et al. 2001; Moczek and Nagy 2005). In *O. taurus*, larval nutrition directly affects pupal (and adult, final) body size (Moczek 1998; Moczek and Emlen 2000) and, at the same time, typical rearing conditions in the lab are such that stochastic variation in dung quality and amount within our experimentally reared animals generates sufficient variation in body size around the threshold for alternative nutritional morph production. Thus, we used body mass, measured at the first day of pupal development, as an estimate of feeding conditions experienced during larval development. Specifically, pupae weighing less than 0.105 g were considered small, low‐nutrition individuals; male pupae in this weight class mature into the minor, hornless morph without exception. In contrast, pupae weighing more than 0.12 g were considered large, high nutrition individuals; male pupae in this weight class uniformly metamorphose into the major, horned morph. Pupae with intermediate weights (0.105–0.12 g) were excluded from the experiment as their developmental fate is difficult to predict. We focused on day 1 pupae because (2) this stage enables us to capture the most important developmental processes underlying late prepupal growth and early pupal differentiation of adult traits, (2) animals can be unambiguously sexed and phenotypes scored (e.g. horned and hornless) and (3) dissections are easy and fast, thereby minimizing chances for RNA contamination and degradation.

### Tissue harvesting and RNA extraction

A total of 64 *O. taurus* first‐day pupae were sacrificed and dissected for microarrays as described in Kijimoto et al. (2014), 16 each for large (high nutrition) horned males; small (low nutrition) hornless males; large females and small females. We dissected epidermal tissue of the developing head horns (or the homologous area for females, which lack head horns) and prothoracic horns, in addition to all six legs and a small section of the dorsal abdominal epidermis (with attached muscle gently scraped off), detailed further in Kijimoto et al. (2009, 2014) and Snell‐Rood et al. (2011). We then created biological replicates by pooling tissue of four individuals in the same tissue–sex–size category.

We used the same individuals in batches across tissue types and subsets of samples were included as technical replicates wherein labeling and hybridization were repeated independently on the same RNA sample.

### Statistical estimation of gene expression levels

We normalized raw microarray measurements to account for within‐array artifacts and to allow comparison of measurements across arrays by (2) eliminating spatial artifacts through fitting a two‐dimensional smoother (loess: (Simonoff 1996); smoothing parameter 0.01) to the red and green channels separately as outlined in the SNOMAD methodology (Parmigiani et al. 2008) and (2) using the Bioconductor package limma (Smyth 2009) to correct for dye‐bias. After within‐array normalization, quantile normalization was used to make measurements across arrays comparable (Bolstad et al. 2003). Using these normalized expression measurements, we estimated differential expression levels among the 16 conditions of interest (two sexes × four tissues × two nutritional states) by Ordinary Least Squares (OLS) as implemented by the lmFit function in the Bioconductor package limma (Benjamini and Hochberg 1995). Limma results were then verified by direct OLS calculations in R. For additional details on the statistical analyses underlying the optimization and execution of this design please see Kijimoto et al. 2014; including the detailed supplemental methods. Further details on this experimental design, its analysis, and its performance relative to more conventional designs are given in Moczek et al. (2014).

### Enrichment analyses

We used eight t‐statistic distributions that characterized the effect of sex on each tissue‐by‐nutrition gene expression set to perform functional enrichment analyses. Briefly, we characterized protein functions based on homology with described genes in the Gene Ontology database (Ashburner et al. 2000) using the program Blast2GO (Conesa et al. 2005) resulting in 1114 functional categories (these categories represent gene product properties, such as their contribution to cell structure, their binding or catalytic activity, or their role in a biological process). Using the program ErmineJ (Lee et al. 2005), we tested each functional category of proteins for an excess of differentially expressed genes relative to all other genes not in that category by using gene score resampling to generate a null distribution.

### Candidate gene analyses

To identify additional candidate genes and pathways underlying condition‐responsive sex‐biased development we compared lists of contigs exhibiting sex bias across tissues and nutritional conditions to putative homologs in other species using BLAST (Tables S3 and S4).

### Comparison of Nutrition‐Dependent and Sex‐Biased contigs

We sought to characterize the repertoire of genes in each tissue that contributes to both sexual dimorphism *and* nutrition responsiveness. Such genes cannot merely be ascertained by identifying contigs in our study that were sex‐biased under one nutritional condition because the behaviors of samples in a microarray analysis are only relative to the behavior of their hybridization partners (Fig. S2). In order to determine whether a sex‐biased gene also changes across nutritional conditions, we found the intersection (commonality) of contigs whose variation in expression levels was significantly explained by sex (detected by hybridizations between male and female individuals under the same condition; this study) and the corresponding pool of contigs whose variation in expression levels was significantly explained by nutrition (detected by hybridizations between high and low nutrition individuals of the same sex; Kijimoto et al. 2014). This comparison was drawn for sex‐biased contigs expressed exclusively in one tissue, the largest class of contigs in our study. To standardize the number of common contigs across sexes and tissues, the intersection of each sex‐tissue group was divided by the union (i.e., the total number of sex‐biased and nutrition‐dependent contigs for that group without repeats) and multiplied by 100 (Fig. 6C).

## Results

In this study, we sought to quantify and contrast sex‐biased gene expression as a function of tissue type and nutrition in the beetle *O. taurus*. Recall that, in this species, sexual dimorphism on a morphological level is most pronounced at high nutrition, and greatest for head horns followed by thoracic horns, but modest for legs, and absent for abdominal epithelium. At low nutrition, in contrast, the same four traits exhibit minimal to no sexual dimorphism on a morphological level (Fig. 1). By comparing genome‐wide sex‐biased gene expression across four tissues and two nutritional conditions, we examined basic hypotheses regarding the developmental‐genetic mechanisms underlying sexually dimorphic and nutritionally sensitive phenotypes.

### Sex bias as a function of tissue and nutrition

To guide our analyses of these data we examined three basic hypotheses regarding the relationships between sex‐biased gene expression and sexual dimorphism evident on a morphological level. First, we predicted a positive correlation between the degree of sex‐biased gene expression and the degree of morphological sex differences. Specifically, we predicted that traits exhibiting drastic sexual dimorphism in morphology (e.g., head horns) should express more sex‐biased contigs than traits that demonstrate subtle or no sexual dimorphism (e.g., legs or abdominal epithelium). Second, and related, we predicted that this pattern would be most evident under the nutritional conditions that promote sexual dimorphism (i.e., high nutrition). Third, we predicted that sex‐biased gene expression should be most frequent in the sex that engages in nutrition‐dependent growth of secondary sexual traits (i.e., males).

**Figure 2:**
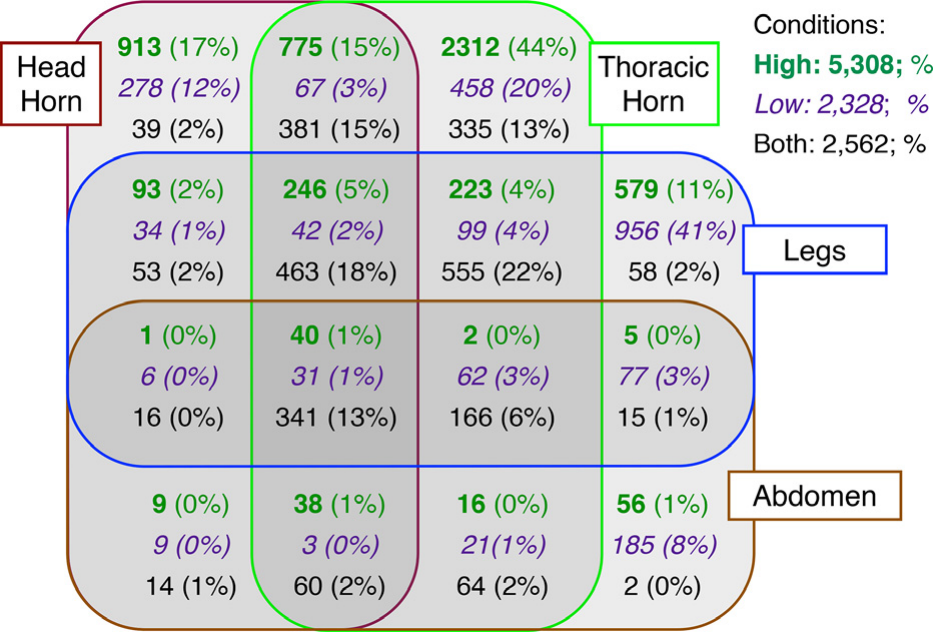
Number of sex‐biased contigs unique to, or shared among, four different tissues. Different colors indicate numbers and percentages of contigs that exhibit sex bias under high (green), low (purple), or both (black) conditions.

**Figure 3:**
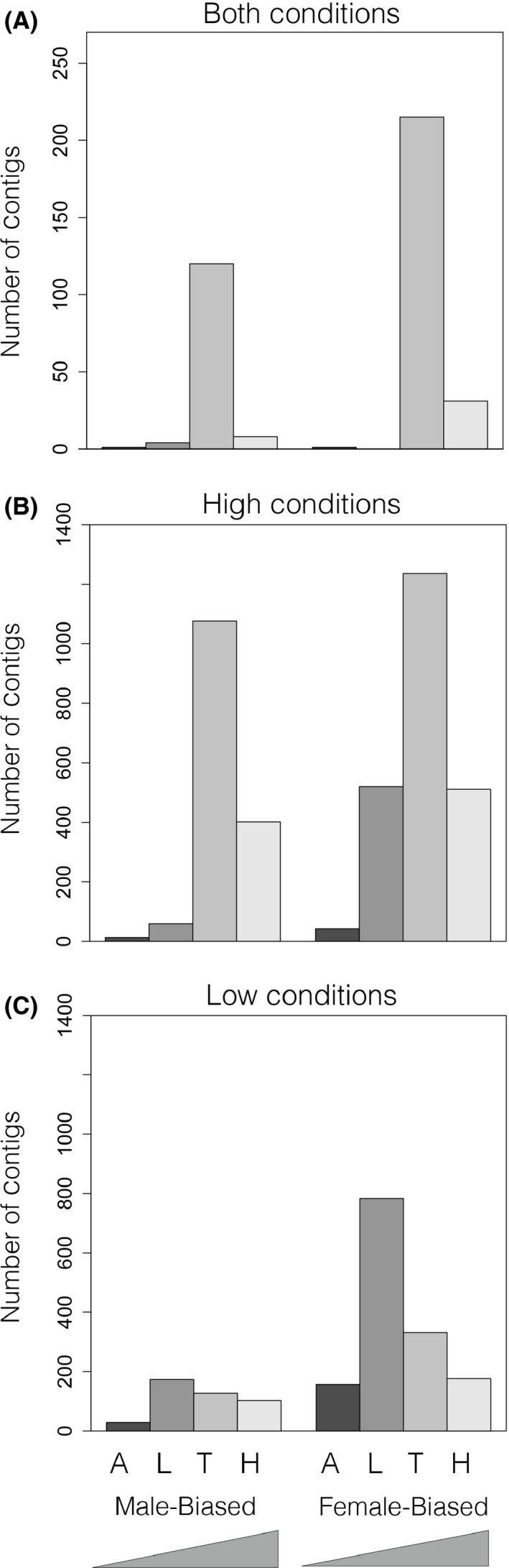
Number of sex‐biased contigs as a function of tissue and nutritional condition. The magnitude of sex‐biased gene expression, for genes expressed exclusively in one tissue, varies across traits and nutritional conditions (A = abdominal epidermis; L = leg epidermis; T = thoracic horn epidermis; H = head horn epidermis). Ramps on *x*‐axis indicate the increasing degree of sexual dimorphism among focal traits.

Our first and second predictions were met, though only in part. We found that sex‐biased contigs were most numerous in head‐ and thoracic horns and least numerous in legs and abdominal epithelium, specifically when considering contigs that were sex‐biased exclusively under high nutrition (bold green numbers in Fig. 2) or contigs who were sex‐biased under both nutritional conditions (black numbers in Fig. 2). For example, under high nutrition, head‐ and thoracic horns exhibited 913 and 2312 sex‐biased contigs, respectively, whereas sex bias was considerably lower in legs (579) and minimal in abdominal epidermis (56). Furthermore, the overall magnitude of sex‐biased gene expression declined with nutrition (italic purple numbers in Fig. 2), but when individual tissues were considered it became clear that this effect was restricted to both horn types. Specifically, while the number of sex‐biased contigs in the head‐ and thoracic horns decline threefold (to 278) and fivefold (to 458), respectively, the sex‐biased contig pool of legs nearly doubled (to 956) and tripled (to 185) for abdominal epithelium. Lastly, our third prediction failed to be met regardless of nutritional conditions: female‐biased contigs were as frequent, or more frequent, irrespective of whether they were nutrition‐dependent or independent, raising the possibility that reducing trait growth in females may require substantial female‐biased gene expression (Fig. 3).

**Figure 4:**
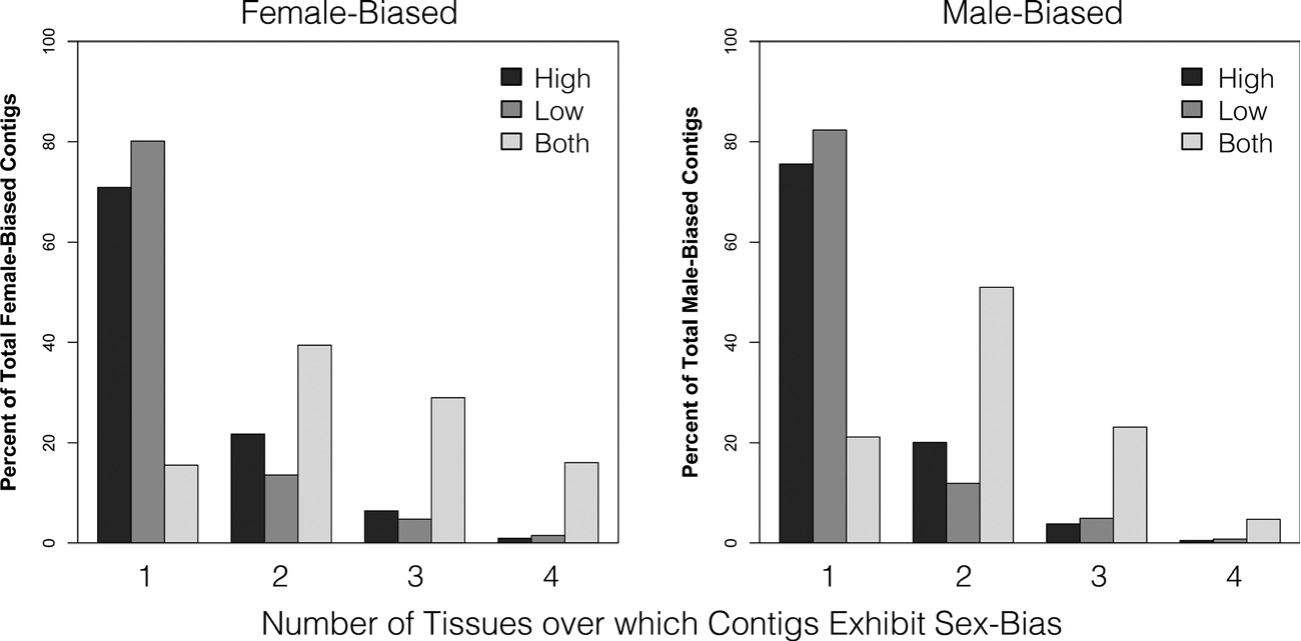
Sex bias as a function of tissue‐specificity and nutrition dependence. The vast majority of sex‐biased contigs that are also condition‐dependent (expressed exclusively under high or low conditions) are unique to one tissue. In contrast, there are as many sex‐biased contigs that are condition‐*in*dependent (being expressed under both high and low conditions) expressed over many tissues as there are expressed in a tissue‐specific manner. This pattern is true for both female‐ and male‐biased contigs.

Next, we sought to determine the extent to which sex‐biased contigs were *unique to* or *shared over* multiple tissues. Theory predicts that genes expressed in many tissues are subjected to stronger pleiotropic constraints (Meisel 2011), thus possessing less freedom to evolve sex‐biased expression; therefore, we expected that sex‐biased contigs expressed in only one tissue would be more prevalent than those expressed in many. Our data partially support this prediction: The vast majority of sex‐biased contigs that also exhibited condition‐dependence (i.e. were sex‐biased exclusively under high *or* low nutrition) were unique to one tissue, and this number decreased with increasing distribution over multiple tissue (Fig. 4). In contrast, there were as many sex‐biased contigs that were condition‐*independent* (i.e., sex‐biased under high *and* low conditions) expressed over multiple tissues as there were expressed solely in one tissue, a pattern that held true for both female‐ and male‐biased contigs.

### Sex‐biased transcriptomes at a functional level

**Figure 5:**
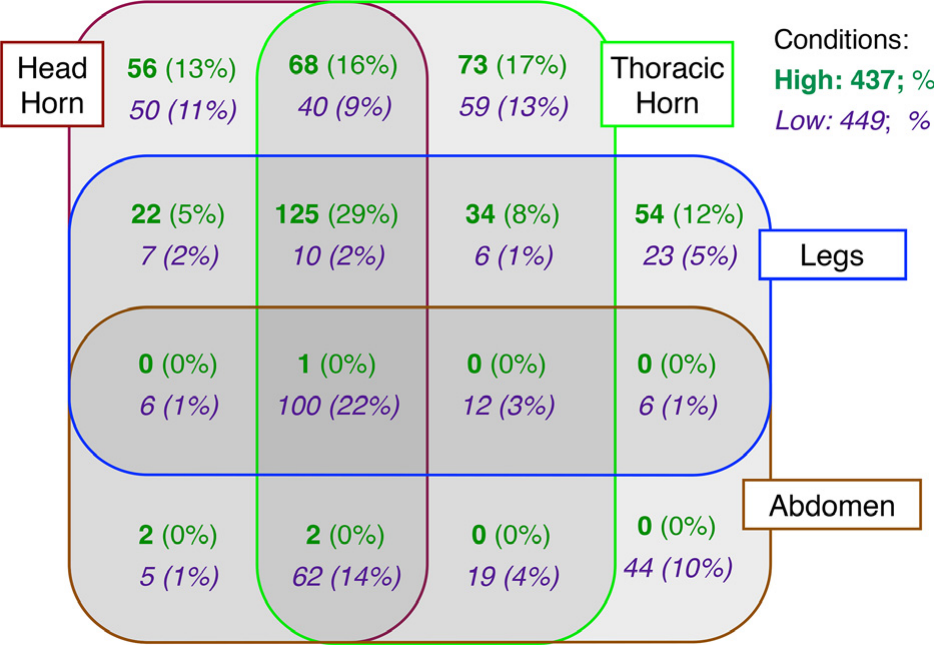
Sex‐biased GO terms overrepresented among four tissues and under high and low conditions. Numbers and percentages of sex‐biased gene ontology (GO) terms overrepresented among four different tissues under high (green), low (purple), or both (black) conditions. Percentages (in parentheses) indicate the fraction of GO terms in a particular tissues or tissue combination relative to the total number of GO terms for a particular nutritional condition (437 and 449, respectively).

To assess functional similarities and differences among pools of sex‐biased contigs in each tissue‐nutrition comparison, we tested for the overrepresentation of differentially expressed functional categories in each of the 1114 Gene Ontology categories represented in our dataset (Table S1). Averaged across all four tissues, transcriptome‐wide sex‐biased gene expression revealed similar numbers of functional categories enriched under high and low conditions; 437 sex‐biased categories exhibiting significant enrichment under high nutrition conditions (39% of the 1114 categories tested, FDR *P* < 0.05) and 449 under low nutrition conditions (40% of the 1114 categories tested, FDR *P* < 0.05; Fig. 5). We asked how these sex‐biased lists of enriched functional categories (for each tissue‐nutrition group) were unique to or shared across tissues; if functional categories derived from independent enrichment analyses (from each tissue) were common across different tissues, this would suggest that independent sex‐biased gene repertoires were converging on similar functional processes.

For both high and low nutritional conditions, many enriched sex‐biased functional categories were found in single tissues (as opposed to being common to two, three, or four tissues), such that the number of functional categories across tissues generally mirrored the number of sex‐biased contigs expressed across those tissues (Fig. 2). This result suggests that, under either nutritional condition, the unique gene pools expressed among traits translate into unique functional processes.

However, a striking pattern that emerged from subsequent comparisons was that sex‐biased functional processes exhibited notable overlap among three and four tissues under high and low, conditions, respectively, even though these tissues express largely independent repertoires of sex‐biased genes. For instance, under low nutrition, although all four tissues share only 1% (31 of 2328; Fig. 2) of sex‐biased contigs, they converged on 22% (100 of 449; Fig. 5) of sex‐biased functional groups. An analogous pattern emerged across three tissues (horns and legs) under high conditions: although these tissues shared only 5% (246 of 5308) of expressed, sex‐biased contigs, they converged on 29% (125 of 437) sex‐biased functional groups. Together, these results suggest that, under either nutritional condition, traits within an individual can converge on similar biological functions irrespective of their underlying gene repertoire.

Finally, the types of functional categories that were enriched for sex‐biased genes varied over tissues and nutritional condition. For instance, in head horns, 27% of categories enriched for sex‐biased genes were related to metabolism or biosynthesis under high nutrition, compared to 4% under low nutrition (Fig. S1, Table S2), mirroring results from previous studies on nutrition‐biased data (Kijimoto et al. 2014). Conversely, head horns demonstrated an abundance (12%) of categories enriched for sex‐biased genes related to development and morphogenesis under *low* conditions relative to high nutrition (4%), suggesting that many sex‐biased developmental processes may still be deployed despite, or perhaps because of, the development of a sexual monomorphism at a morphological level (i.e., under low nutrition). A similar pattern was observed in thoracic horns.

### Sex‐biased and nutrition‐responsive gene expression: similarities and differences

We examined whether the same developmental genetic machinery might contribute to both the sexual dimorphism *and* nutrition dependence of traits, a prediction made by previous theoretical work (Rowe and Houle 1996; Bonduriansky and Rowe 2005). Following the genic capture hypothesis, we predicted that genes generating both sex bias and nutrition dependence would be most numerous in traits under sexual selection, specifically in the sex experiencing sexual selection (i.e., males).

**Figure 6:**
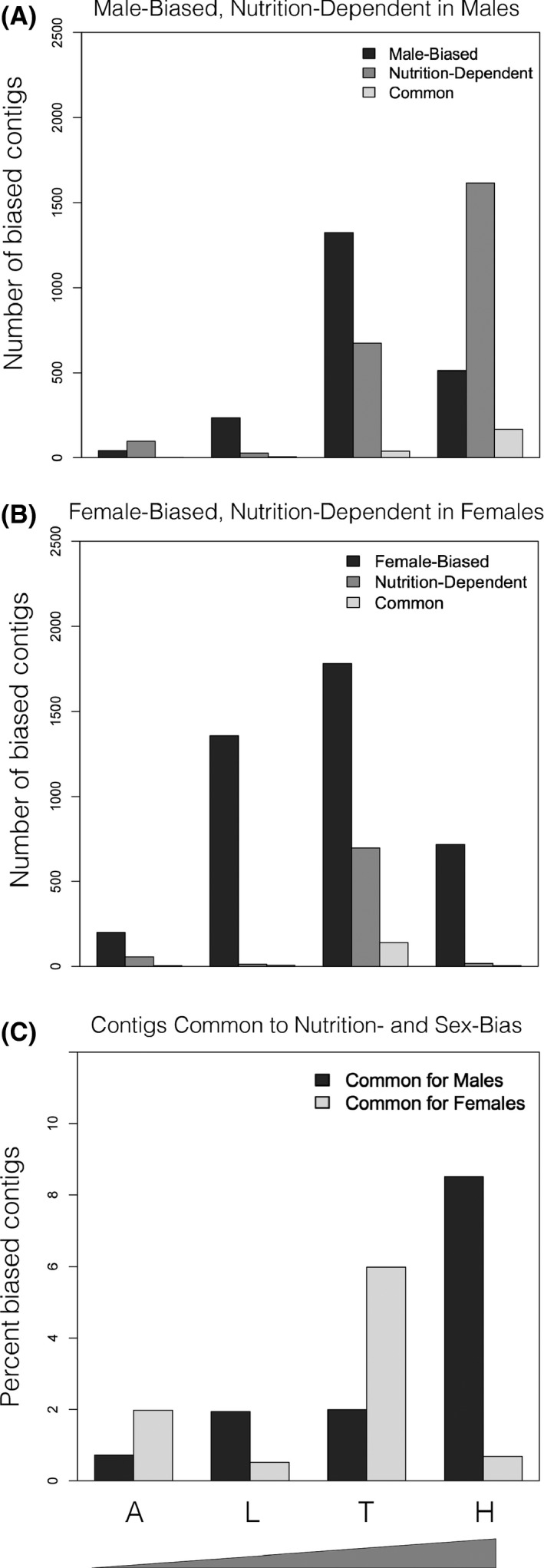
Gene expression common to nutrition dependence and sex‐bias. Sex‐biased contigs are more numerous relative to nutrition‐biased contigs, and this pattern is driven by those with higher expression in females (compare A and B). The number of contigs common to sex bias and nutrition dependence is highest in the trait that demonstrates the highest degree of nutrition‐dependent morphological sexual dimorphism and experiences sexual selection: male head horns (C; percent of the union of sex‐biased and nutrition‐dependent contigs for that tissue). The same pattern to a lesser degree in female thoracic horns (A = abdominal epidermis; L = leg epidermis; T = thoracic horn epidermis; H = head horn epidermis). Ramp on *x*‐axis indicates the increasing degree of sexual dimorphism among focal traits.

In line with predictions from the genic capture hypothesis, we found that the greatest category of contigs common to nutrition and sex bias were those which were male‐biased and expressed in head horns, a sexually selected trait (Fig. 6C). In contrast, the proportion of contigs common to both sex bias and nutrition dependence was uniformly low across all other categories, with the sole exception being female‐biased contigs expressed in the thoracic horn (Fig. 6C).

## Discussion

In this study, we characterized transcriptome‐wide sex‐biased gene expression among four distinct tissues of male and female *O. taurus* under high and low nutritional conditions in order to investigate the developmental genetic underpinnings of nutrition‐dependent sexual dimorphism and its integration across multiple tissues. Characterizing such sex‐biased gene expression, in turn, allowed us to evaluate how gene expression likely relates to biological processes, and the evolutionary outcomes of sex‐, nutrition‐, and tissue‐specific gene expression.

### Sex bias as a function of tissue and nutrition

Previous studies have demonstrated that organisms possess ample sex bias in gene expression, and that the degree and nature of this expression differs among tissues (Yang et al. 2006; Ellegren et al. 2007; Baker et al. 2011; Pointer et al. 2013). Here, we were able to refine and extend this type of research by determining how sex‐biased gene expression is related to sexual dimorphism in morphology and across different nutritional contexts. We found that the number of sex‐biased genes expressed in a tissue was related to a given tissue's potential to generate morphological sex differences and to the nutritional conditions most conducive to the production of morphological sex differences: sexually dimorphic horns expressed a far greater number of sex‐biased genes than the largely sexually monomorphic legs or abdominal epidermis, yet did so only under high nutritional conditions. However, we also detected that while under low nutritional conditions the degree of sex‐biased gene expression decreased in horn tissues, it increased in legs and abdominal epidermis. Thus, reduced sexual dimorphism at a morphological level may coincide with an overall reduction in sex‐biased gene expression, but this pattern is dependent on the tissue in question.

Additionally, we found female‐biased gene expression was as frequent as male‐biased gene expression, regardless of tissue and irrespective of whether gene expression also exhibited nutrition‐dependency. This challenges the notion that female development and morphologies are “baseline” for *O. taurus*, with traits such as large horns reflecting a departure from baseline. Instead, this suggests that female‐specific trait growth, or superficial lack of growth, may require as much female‐biased gene expression as male‐specific trait exaggeration requires male‐biased gene expression. Indeed, among *Onthophagus* species, additional female‐specific remodeling of pupal traits can create sexual dimorphism in initially monomorphic traits, or magnify preexisting sex differences (Moczek 2006).

Another striking pattern observed across sex‐biased genes was that the majority were tissue‐specific, insofar as they were also condition‐dependent (i.e., they were sex‐biased exclusively under high or low nutritional conditions). In contrast, genes that were sex‐biased under both nutritional conditions were as commonly expressed in single tissues as they were across multiple tissues. These results are largely consistent with our prediction that sex‐biased genes would be more prevalent with increasing tissue specificity. This prediction stems from the expectation that genes deployed in several tissues are subject to pleiotropic constraints, which could limit their ability to evolve sex‐biased gene expression (Hodgkin 1998; Duret and Mouchiroud 2000; Mank et al. 2008; Meisel 2011). Thus, if sex‐biased gene expression evolves to resolve the sexual antagonism arising from different phenotypic optima between the sexes (Lande 1980; Rice 1984), it may most readily evolve for genes expressed in fewer tissues.

Together, our results suggest that sexually dimorphic and condition‐dependent phenotypes are comprised of traits underlain by independent, tissue‐specific repertoires of sex‐biased gene expression, and that these traits may therefore evolve independently without correlated responses to selection (Badyaev et al. 2001). This then begs the question: how do developmentally independent traits elicit a coordinated response to varying nutritional conditions to generate a whole and functionally integrated organism?

### Integration of sex‐biased gene expression at a functional level

We sought to determine the extent to which sex‐biased gene expression may result in biological functions that are unique to individual, or shared (i.e., integrated) among multiple tissues, and the dependency of these biological function on nutritional conditions. Because gene products can serve multiple biological functions, and multiple genes can serve the same biological function, different tissues might elicit unique or integrated biological functions irrespective of underlying gene expression patterns.

Under both high and low nutritional conditions, many sex‐biased functional categories were tissue‐specific, suggesting that, under either condition, *O. taurus* males and females reflect, in part, mosaics of sex‐biased functional processes deployed in a tissue‐specific manner. However, sex‐biased functional processes exhibited notable overlap among three and four tissues under high and low conditions, respectively, despite the fact that these tissues express largely independent repertoires of sex‐biased genes, a pattern distinctly different from that observed for nutrition‐dependent genes (Kijimoto et al. 2014). For instance, in individuals experiencing high nutritional conditions, a pool of similar sex‐biased functional responses was shared by head horns, thoracic horns and legs (29% of the total functional terms under high conditions). This may be due to their many shared morphogenetic properties as all three constitute 3‐dimensional epidermal outbuddings, which previous studies have shown rely on a shared set of conserved pathways to regulate growth and axis formation. Likewise, all four tissues shared a surprisingly large proportion of sex‐biased functional processes in individuals experiencing low nutritional conditions (22% of the total number of sex‐biased functional processes enriched over low nutritional conditions), despite the fact that only 1% of sex‐biased contigs were expressed across all four tissues. Because enriched functional processes were shared across tissues yet derived from tissue‐*specific* gene repertoires, our data suggest that individual tissues are *converging* on similar functions in a sex‐biased and nutrition‐dependent manner, in spite of their underlying, divergent transcriptional profiles.

Importantly, our results revealed an unexpected property regarding the relationship between plastic trait integration and pleiotropy. It has been proposed that integrated phenotypes that serve similar functional and developmental properties are underlain by a common genetic system with pleiotropic effects (e.g. Cheverud 1996; Wagner 1996). In contrast, our results reveal that integrated sexually dimorphic responses to varying nutritional conditions arise in *O. taurus* through independent genetic modules converging on the same biological and developmental functions. If this pattern is a general property of phenotypic responses to nutrition, the diversification of sexually dimorphic phenotypes might be more labile and less subject to pleiotropic constraints than previously thought; each tissue that comprises a sexually dimorphic phenotype, or a nutritional morph within a sex, might act as its own selective target, yet converge on the same functionality as other tissues within the phenotype. That pleiotropic constraints are weakened between modules is not a new concept itself (Raff 1996), but we find here that this also applies to modules deployed in a sex‐ and environment‐specific fashion (Snell‐Rood et al. 2010).

Lastly, we found that the types of sex‐biased functional processes used across tissues and nutritional conditions varied widely. Unsurprisingly, head horns, which demonstrate highly divergent sex‐biased growth patterns under high nutrition, employ a great number of sex‐biased biological processes related to metabolism and biosynthesis under high conditions. Interestingly, they also employ an abundance of sex‐biased biological processes related to development and morphogenesis under low conditions, corroborating a recurring theme that achieving sexual *monomorphism* at a morphological level might, too, necessitate the recruitment of diverse developmental processes (e.g., negative growth regulators or programmed cell death).

### Is there a common developmental genetic basis for sexual dimorphism and nutrition dependence?

We examined whether, and for what traits, sexually dimorphic development may be underlain by the same developmental‐genetic processes that also enable condition‐dependent development. Such an overlap is predicted by sexual selection theory (Rowe and Houle 1996; Kotiaho et al. 2001; Bonduriansky and Rowe 2005; Bonduriansky 2007), and specifically the genic capture hypothesis, which posits that sexually selected traits should evolve condition‐dependence. In this scenario, a male‐limited modifier that shifts the growth of a secondary sexual trait towards higher values in higher condition males, relative to lower condition males and females, results in an optimal sexual dimorphism. However, this mechanism of genic capture depends on the existence of modifiers that are both sex‐limited and condition‐dependent, which has limited empirical validation (Bonduriansky and Chenoweth 2009). Thus, a key prediction of genic capture is that gene expression underlying sexually selected traits should associate not only with sexual dimorphism but also condition (Tomkins et al. 2004), a prediction largely supported by our results.

Specifically, we found that while the contig repertoire exhibiting both nutrition dependence and sex bias in *O. taurus* was generally small, it was greatest in the category of contigs that were male‐biased and expressed in head horns, the tissue and sex that is sexually selected in this species. This observation supports the expectation that the evolution of condition‐dependent sexually selected traits can be facilitated by a modest set of genes that directly influence both; specifically, it appears that male‐limited and condition dependent modifiers contribute to gene expression underlying qualitative exaggeration of head horns in *O. taurus*.

### Candidate pathways for generating sexual dimorphism

Our analysis identified diverse candidate genes and pathways possibly underlying the regulation of sexually dimorphic development, including pathways previously functionally implicated in the development of sexual dimorphisms such as the transcription factor *doublesex* and the *hedgehog‐*receptor *patched* (Kijimoto et al. 2012; Kijimoto & Moczek still in review). Other identified genes, such as several heat shock proteins, have been shown to exhibit sex‐biased expression in species besides *O. taurus*, such as the silkworm *Bombyx mori,* yet their function with respect to sexual dimorphism remains to be characterized (Xia et al. 2007). Moreover, several candidate genes revealed by our study have been previously identified as sex‐related, but usually in the context of reproductive tissue development. For instance, in *Drosophila*, angiotensin‐converting enzyme (*ance*) is known for its role in spermatogenesis (Hurst et al. 2003), yet in this study was found to be female‐biased across all tissues. Similarly, the gene hu li tai shao (*hts*) has been implicated in filament growth at the oocyte cortex and assembling actin at ring canals in developing egg chambers (Yue and Spradling 1992; Pokrywka et al. 2014), yet in this study was found to be male‐biased across all tissues.

Lastly, our study identified as sex‐biased certain categories of genes whose functions are well understood in other physiological contexts, but have not previously been implicated in the developmental genetic regulation of sexual dimorphism. For instance, circadian rhythm genes such as *cryptochrome*,* disco*, and *rrp12* (Yoshii et al. 2004; Zhang et al. 2009) were male‐biased in head and thoracic horns. In contrast, several neurotransmitters, neurotransmitter receptors or modulators of their metabolism such as catecholamines up (*catsup*), acetylcholine receptor, glutamate receptor interacting protein, and oxytocin receptor were female‐biased in head and thoracic horns. These putative mechanisms for sexual differentiation will provide compelling substrate for future functional studies.

## Conclusions

Our study demonstrates that size, composition, and integration of the gene repertoire responsive to sex are heavily influenced by the identity of the tissues under consideration and the nutritional conditions under which they develop. Some of these interdependencies match intuitions and theoretical predictions, while others were surprising.

First, we found that, as expected, many more sex‐biased genes were expressed under high nutritional conditions – the context where sexual dimorphism at a morphological level is most evident – relative to low nutritional conditions. Furthermore, of these, the vast majority of sex‐biased genes were expressed in a trait‐specific manner, and again following expectations, this diversity generally scaled with the developmental potential of a given trait to engage in sexually dimorphic developments. However, we also observed for at least two traits ‐ legs and abdominal epithelium ‐ that the diversity of sex biased genes actually *increased* under low nutritional conditions (when sexual dimorphism is absent), and that, across all traits, female‐biased expression was as prevalent as male‐biased expression, even though females do not “develop” exaggerated traits. This trend was recapitulated at the level of functional processes: under low conditions, where morphological sexual dimorphism is lacking, numerous sex‐biased functional categories were deployed in horn tissues. Combined, these observations suggest that morphological responsiveness to sex and nutrition is at best a partial guide to the underlying transcriptional dynamics, and that to create sexual dimorphism in development requires the remodeling of both sexes, regardless of which sex exhibits externally visible trait exaggeration.

Our work also suggests that individual tissues can elicit unique repertoires of sex‐biased genes and, at the same time, converge on shared functional processes in different nutritional contexts. This finding has important implications for the evolvability of sexually dimorphic phenotypes: if each tissue possesses its own genetic mutational and selective target, yet is able to converge on the same functionality as other tissues within the same sexual phenotype or nutritional morph within a sex, then these tissues are free to diversify among lineages in their sex‐ and nutrition‐responsiveness relatively independent of each other. It is tempting to speculate that these evolutionary degrees of freedom may have contributed to the massive radiation of *Onthophagus* into over 2000 extant species with varying degrees of trait exaggeration and sexual dimorphism (Moczek 2010).

Our study also provides evidence in support of two evolutionary predictions. First, the vast number of sex‐biased genes were expressed in only one tissue, consistent with the prediction that sex‐biased expression is most easily achieved in genes that exhibit tissue‐limited expression and are therefore less pleiotropically constrained (Mank et al. 2008; Meisel 2011). Second, our study provided insight into the common developmental genetic basis of nutrition‐dependent and sexual dimorphism predicted by condition‐dependence theory (Rowe and Houle 1996; Bonduriansky and Rowe 2005; Bonduriansky 2007). Even though only a modest proportion of sex‐biased genes were also strongly implicated in nutrition dependence, this pool of genes was concentrated in a sexually selected tissue in the sex experiencing sexual selection, consistent with predictions.

Lastly, our study has enriched the pool of candidate genes and pathways that may underlie sex‐specific trait expression and its modulation by nutrition. Given the transcriptomic, genomic, and gene‐function tools now available in *Onthophagus* on one side, and it richness in plasticity and sexual dimorphism over a range of phylogenetic distances on the other, these findings create the opportunity to address the next suite of critical question in the evolution and development of condition responsiveness: Do nutrition‐dependent genes arise from sex‐biased genes, or *visa versa*? Do genes with tissue‐specific expression evolve sex‐bias, or do sex‐biased genes evolve tissue‐specificity? Which functional characteristics are most conducive to, or resistant against, conditional activation and integration in developmental evolution?

## Data Accessibility

Array data: NCBI GEO accession number GSE58496. SNP data: DRYAD accession number doi:10.5061/dryad.gr5n3.

## Supporting information


**Figure S1.** Sex‐biased, enriched functional categories differ over body regions and conditions when considering those that are sex‐biased exclusively in those tissues.

**Figure S2.** Cases represent different theoretical expression behaviors of genes in different sex and nutritional contexts.

**Table S1.** Gene ontology (GO) terms significantly enriched (FDR < 0.05) among sex‐biased genes expressed in head horn, thoracic horn, leg, or abdominal epidermis in either large or small *O. taurus,* regardless of overlap between tissues.

**Table S2.** Functional categories enriched exclusively in head horn, thoracic horn, leg, or abdominal epidermis in either large or small *O. taurus*.

**Table S3.** Genes identified by NCBI BLAST that correspond to contigs that were sex‐biased and female‐biased across head horns, thoracic horns, legs, or abdominal epidermis.

**Table S4.** Genes identified by NCBI BLAST that correspond to contigs that were sex‐biased and male‐biased across head horns, thoracic horns, legs, or abdominal epidermis.
